# Severe HAPE in a Remote High-Altitude Research Station in Antarctica

**DOI:** 10.3390/life16020313

**Published:** 2026-02-11

**Authors:** Fanny Larcher, Paul Laforet, Stephane Fraize, Mario Lecca, Riccardo Scipinotti, Gianluca Bianchi Fasani, Salvatore Vagnoni, Sascha Freigang

**Affiliations:** 1 French Polar Institute Paul-Émile Victor (IPEV), 29280 Plouzané, France; 2 Terres Australes et Antarctiques Françaises (TAAF), 97410 Saint-Pierre, France; 3 Programma Nazionale di Ricerche in Antartide (PNRA), 00185 Rome, Italy; 4 European Space Agency (ESA), French Polar Institute Paul-Émile Victor (IPEV), Department of Neurosurgery, Medical University Graz, 8010 Graz, Austria

**Keywords:** HAPE, mountain disease, isolated medicine, polar medicine, emergency medicine, hyperbaric chamber, high altitude pulmonary oedema, altitude illness

## Abstract

Isolated, confined, and extreme environments hold the opportunity to collect unique biomedical data. These often-remote places present specific medical challenges for deployed expeditioners. Here we report a case of acute severe high altitude pulmonary oedema (HAPE) and its management at a remote research station in Antarctica. At the beginning of the 2023 summer campaign at Concordia Station (3200 m AMSL), a technician presented with shortness of breath and compromised circulation three days after arrival on site. Immediate diagnostics and medical treatments with high-flow oxygen and the use of a mobile hyperbaric chamber after initial resuscitation were administered. Within a time window of 24 h, evacuation to sea level was organised via aircraft (flight duration 4 h, non-pressurised cabin) inside the mobile hyperbaric chamber. The patient was discharged from medical treatment 48 h later in Christchurch (NZ). We conclude that despite rigorous pre-deployment screening, even experienced expeditioners can develop critical medical conditions that require prompt reaction and rescue. Structured assessment tools can aid in their recognition and management.

## 1. Introduction

**Figure 1:**
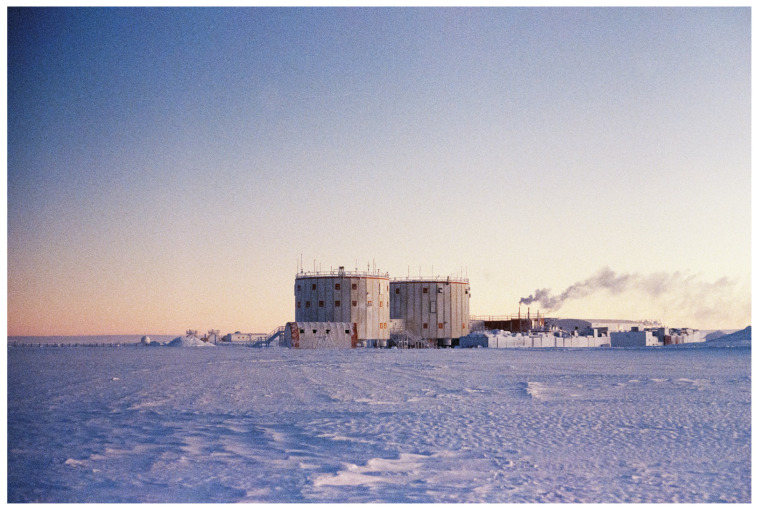
Main Towers of Concordia Research Station as seen from the summer camp.

**Figure 2:**
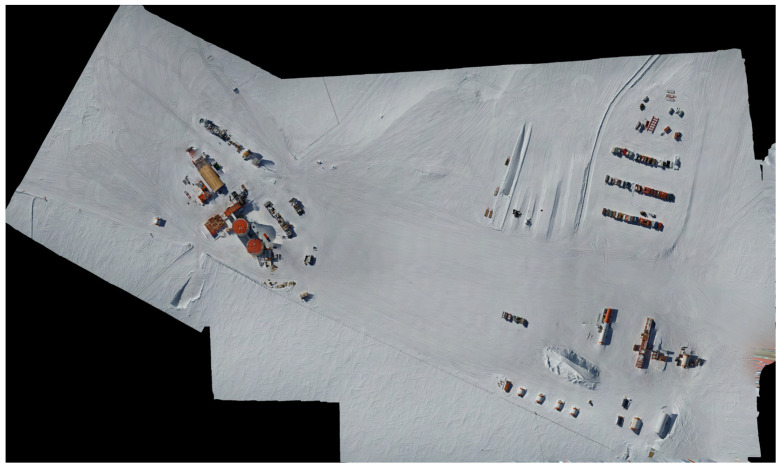
Drone Image of Main Towers on left side and Summer Camp on bottom right side; the rescue was conducted from the tents seen on the bottom right side (5 white squares) to the Main Towers.

The pathophysiological processes of diseases associated with high altitude are subject to ongoing research. Often, they are preceded by a pattern of alterations in pulmonary mechanics and gas exchange [1,2,3,4]. Main risk factors include fast ascent to altitude and high exertion levels [3,5,6]. The Concordia research station is located on an Antarctic plateau called Dome Charlie at 3233 m AMSL (75°06′ S–123°21′ E, Figure 1 and Figure 2). The atmospheric pressure at Concordia can reach equivalent altitudes up to 3800 m AMSL, and the majority of people arrive by plane. Therefore, acute mountain sickness (AMS) is a very common experience for many Concordia expeditioners during the first days after arrival. Through advised rest and clinical monitoring, the medical team tries to prevent severe cases of high-altitude pulmonary oedema (HAPE) or high-altitude cerebral oedema (HACE), in order to avoid a complicated and dangerous evacuation. The standard evaluation at Concordia station for arriving expeditioners includes assessment of vital signs and safety instructions to rest for 48 h, as well as to present to the medical area for any symptoms, e.g., refractory headache and shortness of breath.

## 2. Case Presentation

Here, we report about a 34-year-old male with non-significant medical history, who arrived at Concordia station two days prior to symptom onset. At the time, he had extensive local experience and had spent several summer campaigns at Concordia within the last 3 years. Shortly after his arrival, he performed tasks of high physical demand by removing snow from the shelters and containers, and exertion was facilitated by frequently going up and down many flights of stairs inside the station. Approximately 48 h after his arrival, he presented to the hospital area of Concordia with a headache. His vital signs were as follows: SpO_2_ of 79%, RR 18/min, HR 90/min. He was treated with paracetamol 1 g and 30 min of oxygen 4 L/min via nasal cannula. No prior medication or other treatments were noted at the time. Subsequently, his SpO_2_ increased to 84%, and he felt subjectively better.

The next day (~72 h after arrival), he did not attend the morning meeting or lunch. His roommates reported continuous nocturnal coughing from the patient. As he was sleeping in shelters located outside the station approximately 500 m away, subsequently, an evacuation to the hospital was initiated. When found by the rescue team, the patient was leaving the dormitory tent close to the Concordia summer camp and was helped into the Pistenbully vehicle to transport him to the station. Initially, he was maintaining his own airway, presented dyspneic with a RR of roughly 25/min, a SpO_2_ of 81%, and a HR of ~120/min, and his skin looked pale without peripheral or central cyanosis. He was oriented, GCS 15, and able to speak single words, limited by respiratory exhaustion. Auscultation and measurement of blood pressure were impractical and omitted due to heavy polar clothing. The patient received 15 L/min high-flow O_2_ via a facemask and was transported in a sitting position to the station. At the time, he was wearing jeans, polar boots, and a polar-gear jacket (Marck & Balsan, Gennevilliers, France). The evacuation took around ten minutes in total.

**Figure 3:**
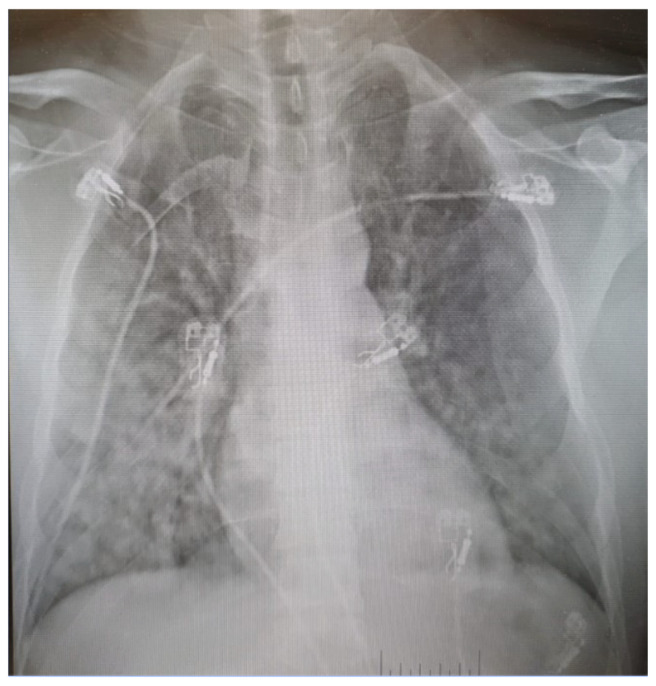
Initial assessment chest X-Ray.

On arrival at the hospital area, he was confused and complained of a frontal headache. The SpO_2_ was 55% after the transfer to the ICU bed, and his RR was 46/min with the presence of a dry cough. The auscultation showed a crackling noise in both lungs. The blood pressure was measured at 155/110 mmHg symmetrical, and the ECG showed a sinus rhythm with tachycardia at a HR of 160/min. The rest of the clinical examination was normal. A severe high altitude pulmonary oedema was suspected. He received corticosteroids intravenously and was put on 15 L/min of oxygen with a high-flow facemask. A pulmonary X-ray concurred with our diagnosis: right dominant, yet bilateral multifocal asymmetrical pulmonary opacities with patchy distribution. Additionally, focal areas of confluence with some well-defined, rounded opacities were noted (Figure 3).

After two hours of high-flow oxygen administration (15 L/min), the patient appeared less confused, and his HR decreased to ~120/min. We introduced Nifedipine 20 mg slow-release capsule and prepared a session in the mobile hyperbaric chamber (Certec ^®^, 69210 Sourcieux les Mines, France). Initially, a 1 h session at full use according to the handbook (interval venting) on 220 mbar was conducted. The patient remained off the chamber until the evacuation flight. CPAP, although available, was not utilised due to limited oxygen and pressurised air supply at the station. In parallel, the station leader was organising the medical evacuation to sea level.

**Figure 4:**
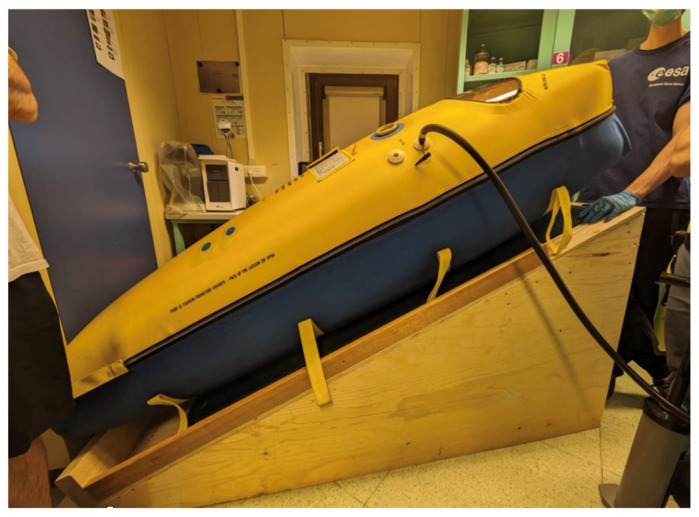
Mobile hyperbaric chamber on Pentetto.

As soon as the patient went into a supine position in bed, his SpO_2_ level deteriorated, and he became dyspneic. Consequently, we decided to build a slope named Pentetto, which enabled a reversed Trendelenburg position of the patient inside the portable hyperbaric chamber (Figure 4).

After one hour in the hyperbaric chamber, the following vital signs were noted: HR 92/min and SpO_2_ of 92% at 5 L/min oxygen via nasal cannula. Given this temporary recompensation, the situation allowed us to wait for the evacuation outside of the hyperbaric chamber for the rest of the night.

**Figure 5:**
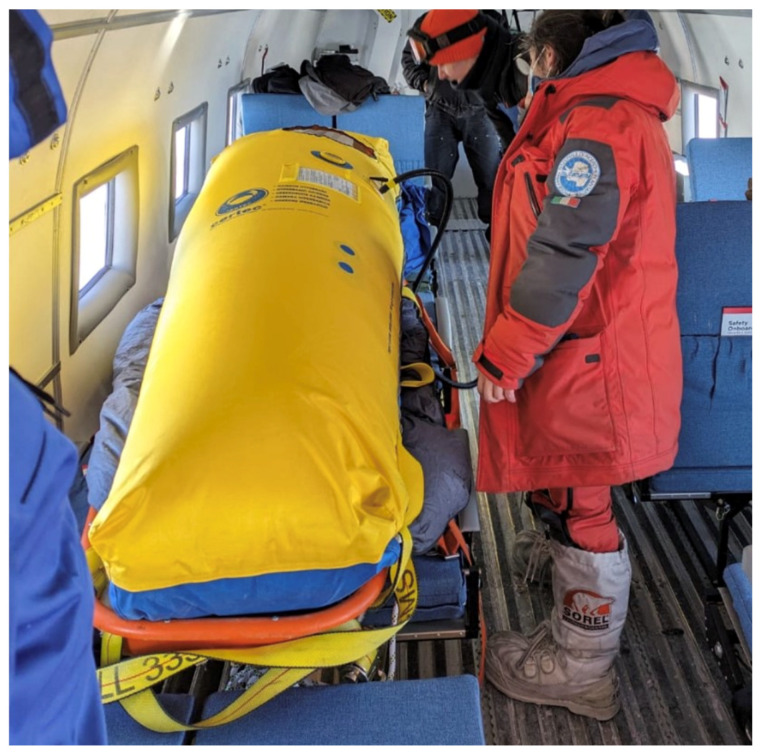
First flight installation (Basler flight from Dome Charlie to Mario Zucchelli Station).

The following day at 8:00 a.m., a Basler DC-3 aircraft arrived at Concordia Station for the medical evacuation. This aircraft type does not have a pressurised cabin; therefore, the patient was placed inside the hyperbaric chamber on the plane for the whole time (Figure 5) and was accompanied by the departing Winter-Over 2023 doctor. A 490 L oxygen cylinder was placed inside the mobile hyperbaric chamber and set to a flow rate of 4 L/min via nasal cannula. The only monitoring device was a pulse oximeter, which could be seen through the chamber window. No verbal communication was possible due to the ambient noise of the aircraft and the sound isolation of the mobile hyperbaric chamber. Therefore, communication was limited to gestures and written notes through the chamber window.

The medical evacuation was uneventful, and SpO_2_ remained above 99% during the flight. The O_2_ cylinder, which was placed inside the mobile hyperbaric chamber, emptied after 2.5 h with no impact on the patients’ vital signs. Upon arrival at the Italian Mario Zuchelli Station around 12 p.m., the hyperbaric chamber was opened, and the patient was placed on 8 L/min O_2_. He was able to walk to the ambulance waiting on the airfield and was brought to the hospital inside the station. Although improved, the patients’ condition did not recover to normal physiology: the O_2_ flow via cannula was reduced to 3 L/min and then 2 L/min with the SpO_2_ remaining above 97%, HR 90–96/min, and a RR of 14/min. At the time, there were no laboratory capacities to advance diagnostics to exclude any underlying conditions, such as cardiac failure or pneumonia. After a telemedical discussion with the TAAF medical chief (Terres australes et antarctiques françaises), the decision to evacuate the patient to New Zealand was made. The same day, an Italian transcontinental military flight was scheduled for 10 pm local time and brought the patient to Christchurch. The second flight was also uneventful, and 2 to 4 L/min of oxygen was administered. 

Upon arrival in New Zealand, the medical evacuation team attended an emergency room, where the patient received 2 L/min of oxygen via nasal canula. The following laboratory results were documented: haemoglobin 14.1 g/L, haematocrit 0.42%, platelets 188 G/L, WBC 12.5 G/L, Na 139 mmol/L, K 4.3 mmol/L, creatinine 69 µ mol/L, EGFR 117 mL/min, CRP 60 mg/L, troponin us 9 mmol/L, blood gas pH 7.43, pO_2_ 71 mmHg, pCO_2_ 36 mmHg, HCO_3_ 23.8 mEq/L. A chest X-ray showed opacities, more pronounced on the right side than the left, consistent with mild-moderate pulmonary oedema. He was transferred to a General Medicine Ward and discharged the following day. Six days after his discharge, he had fully recovered and was able to go hiking as usual without any symptoms.

A full clinical and paraclinical examination in Chamonix, France, was performed six months later by a Medical Doctor specialising in Mountain and High-Altitude Medicine, which revealed no comorbidities that could explain the HAPE episode. No cardiologic nor respiratory conditions, in particular no patent foramen ovale, were found. Echocardiography showed no further abnormalities.

## 3. Discussion

The physiopathology of mountain sickness is not yet perfectly understood. However, HAPE seems to be a form of hydrostatic pulmonary oedema with altered alveolar-capillary permeability [4]. Therefore, exposure to high altitude would lead to vasoconstriction of the pulmonary arterioles and increase the pressure inside the pulmonary capillaries [2]. Also, the vital functional capacity was found to be reduced at altitude, which could be explained by a secondary reduction in muscular compliance due to hypoxia [7]. Hypoxia and an increase in capillary permeability are associated with interstitial oedema [3,4,8]. The interstitial fluid accumulation in lung tissue could induce reduced compliance and a decrease in respiratory muscle strength, resulting in an uneven distribution of ventilation and gas exchange impairments [7]. In the case of our patient, after full recovery and examination, it can be assumed that the cause of HAPE was physical exertion after a rapid, unacclimatized ascent. When a rapid ascent is not avoidable, e.g., arriving at Concordia by plane, it seems that expeditioners need to be at rest for at least 48 h after arrival, to prevent HAPE, AMS, and HACE [6,9].

In general, cold and arid environments, such as research stations within mainland Antarctica, seem to show a higher incidence of AMS than compared to exposure to altitude in moderate climates [10]. The main difficulty at Concordia is the limited accessibility due to its location on a high plateau in the middle of Antarctica, causing many expeditioners to arrive by plane without sufficient acclimatisation. However, we know that normally the ascent to higher altitudes needs to be progressive to avoid these classic mountain illnesses due to hypobaric hypoxia [5]. While HACE often follows worsening symptoms of AMS, HAPE usually develops without precipitating factors and thus presents critical challenges in remote places due to unpredictability [11,12]. A consensus exists that physical exertion significantly contributes to the development of HAPE [5,12,13,14], explaining the need for strict limitation in remote places such as Concordia. In our case, high exertion led to early diagnosis of moderate AMS, progressing to an acute presentation of HAPE. Especially the overlap of classic AMS symptoms during the absence of severe impairment can challenge early recognition of HAPE and present potential diagnostic pitfalls. Reports of nocturnal coughs in combination with previous attendance in the medical area should trigger early assessment. Data on escalation strategies are currently limited, and management plans are even more difficult under the questioned specificity of the Lake Louise Score, which may not sufficiently monitor disease development and severity [15]. The use of systems such as the National Early Warning Score (NEWS) could be helpful in identifying medical problems early [16]. In addition to the clinical picture, laboratory tests and chest X-Ray, non-invasive ultrasound assessment could be used to aid the diagnosis of HAPE [1,17]. We note that in our case report, non-cyanosis was mentioned during the primary survey. We attribute this to the bright lighting conditions at the station during the first assessment outside, peripheral vasoconstriction as a result of the cold environment, leading to a reduced cutaneous blood flow, masking cyanosis, and the heavy polar gear worn by the patient during the rescue period [18,19,20].

This case report adds to a small number of previously published cases of HAPE in Antarctica. Rose et al. [21] reported three cases of expeditioners suffering from HAPE at Amundsen-Scott Station at 2835 m AMSL, whole seven patients had to be evacuated during one season only. Nowadly et al. [22] presented two cases of HAPE, specifically investigating the use of chest X-Rays correlated to disease severity. The reported cases presented within a timeframe of 4 days and SpO2 ranging from 41% to 90%, which is in keeping with our observation. Overall, the incidence of AMS seems to affect 52% of staff arriving at Amundsen-Scott station [10]. For HAPE, prevalence ranges between 0.2% and 15% for expeditioners ascending to elevations above 2000 m AMSL (up to 5500 m), with emphasis on a reverse correlation between period of ascent and likelihood of occurrence, as well as for altitude itself [12,23]. Key contributing factors include rapid ascent, altitudes above 2500–3000 m AMSL, prior history of HAPE, genetic predisposition, structural conditions such as open foramen ovale or pulmonary artery atresia, exertion, cold exposure, and concurrent respiratory infections [14,24,25,26]. Furthermore, the use of oral contraceptives was associated with a higher risk for AMS, and it is reasonable to assume it might also predispose to HAPE [27].

To mitigate the hazards of arriving at remote locations such as Concordia, pre-acclimatisation strategies could be employed to prepare expeditioners. Following expert advice and guidelines, a stepwise reduction in oxygen concentration during the nights preceding the deployment can significantly decrease risks associated with rapid, unacclimatized ascent [28,29]. Financial and logistic burden are within reasonable limits as tents and oxygen generators could be used at home. Experts advised the following altitude profile for pre-acclimatisation in Concordia: first night at 15.5% O_2_ (corresponding to ~2500 m), two nights at 14.5% (~3000 m), and probably (just to improve safety even more) one night at 13.5%, with reference to the experience by Küpper et al. [29]. Regardless of preparation efforts, prompt response to AMS and HAPE is required; in Table 1, we summarize a management plan for Concordia station. Additionally, early intervention with immediately available aids is advised, e.g., Tannheimer et al. have shown significant improvements in SpO_2_ by using simple means such as auto-PEEP [30].

Often summarised as ICE, standing for Isolated Confined and Extreme environment, the weather conditions are extreme and result in significant physiological adaptation processes in the human body. At sea level, the barometric pressure is around 1013 hPa, and oxygen saturation is normally around 98% in healthy individuals. In Concordia, even after many months spent at the base, SpO_2_ rarely increases above 90% at rest. Comparison is often made to a desert-like climate on the basis of its mean annual precipitation. This falls into the ‘hyper arid’ category, shared with the Sahara, Namib, Atacama, and other great deserts of the world. As mentioned earlier, the incidence of AMS seems to be higher in cold and arid conditions [10]; thresholds for symptom monitoring or admission to the medical area should be lowered as a precaution to acknowledge the differences between “Physical altitude” and “Pressure Altitude”. Pressure altitude is the single most important variable of changes in physiology at high altitude [31].

On a separate note, technical factors should be flagged when using pressurised oxygen cylinders in mobile hyperbaric chambers, as this can result in a significant fire hazard. Experts in the field usually recommend strict avoidance of common expedition clothing as it often contains plastic content. This can lead to friction and subsequent electrical charging potential sparks and ignition, especially during the use of an O_2_-source. Expert advice includes jeans and a T-shirt, or wool underwear and blanket only, among the safest options to use in a mobile hyperbaric chamber. Another practical aspect is the limited oxygen supply in remote places and during repatriation flights. Interval administration may extend the duration of increased FiO_2_.

**Figure 6:**
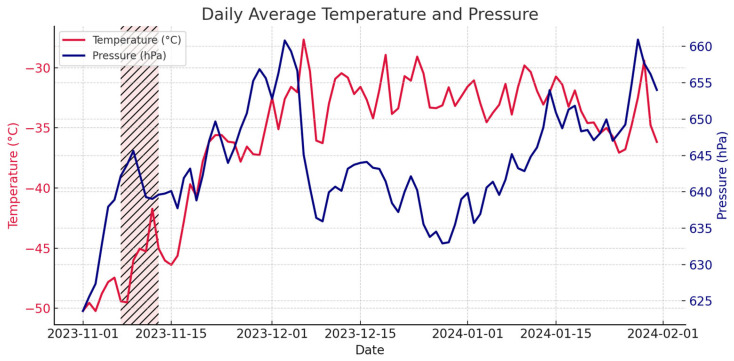
Overview of temperature and atmospheric pressure during the summer campaign at Concordia, the marked area depicts the period of the incident.

For reference, Figure 6 shows the pressure and temperature data from November to February (time window of the summer campaign at Concordia) and the week when the underlying case occurred.

## 4. Conclusions

In the peculiar and remote environment of Concordia, the prevention and management of mountain diseases must be considered carefully. Medical aptitude and awareness of the risks are essential to mitigate risk to the expeditioners.

For the majority of Concordia expeditioners, arrival at Dome C by plane cannot be avoided. Therefore, prevention of mountain diseases is paramount and should include avoidance of physical work for at least 48 h after arrival. Close monitoring of the arriving staff within this critical period seems mandatory. Where available, pre-acclimatisation strategies should be used.

Due to the weather and geographic conditions at the Concordia station, a medical evacuation is sometimes impossible for many days, even during the Antarctic summer period. If descent to sea level cannot be achieved immediately, the use of a mobile hyperbaric chamber, administering additional oxygen, seems to be the best treatment to improve the conditions of patients with HAPE quickly in remote areas.

Following the event, the management plan at the station was updated according to the information provided in Table 1. The stock of pressurised oxygen was increased to enable CPAP treatment and longer field care if needed.

It is important that we continue to study early monitoring of mountain disease to improve prevention and care at high altitude locations in Antarctica.

## Figures and Tables

**Table 1:** DC protocol prevention and care of mountain disease.

Diagnosis	AMS/HACE			HAPE
	AMS		HACE	
**Symptoms**	headache + 1 symptoms (nausea/vomiting, fatigue, lassitude, dizziness)		Moderate-severe AMS symptoms + neurologic sign: ataxia, severe lassitude, altered mental status, encephalopathy	Dyspnoea, non-productive cough, fatigue, weakness, and fever are possible.
	Mild AMS: All symptoms of mild intensity	Moderate-severe AMS: All symptoms of moderate-severe intensity		
**Lake Louise AMS Score**	3–5	6–12	NA	NA
**Differential** **diagnosis**	Migraine, dehydration, hyponatremia, viral infection	Migraine, dehydration, hyponatremia, viral infection	Neurologic stroke, hypoglycemia, severe dehydration, hyponatremia, viral infection	Pneumonia, asthma, bronchospasm, pneumothorax, embolism, heart failure, viral infection
**Prevention**	ACETAZOLAMIDE 125 mg/12 h—DEXAMETHASONE 2 mg/6 h			NIFEDIPINE 30 mg/12 h
	48 h with no physical exercise after arrival in DC			
**Treatment**	PARACETAMOL/ ACETAZOLAMIDE 250 mg/12 h	PARACETAMOL/ ACETAZOLAMIDE 250 mg/12 h	DEXAMETHASONE 8 mg and then 4 mg/6 h (oral, IM or IV)	NIFEDIPINE 30 mg/12 h TADAFIL (10 mg/12 h) or SILDENAFIL (50 mg/8 h) if N. Contradicted
**Oxygen**	No need (*Or if Sp02 < 80%*)	For Sp02 > 90% (1–2 L/min > 2 h)	For Sp02 > 90% Consider a hyperbaric chamber	For Sp02 > 90% Consider a hyperbaric chamber + Pentetto, CPAP, EPAP
**Surveillance**	Daily	Hospital visits twice a day/sleep in base	Hospitalisation— Consider evacuation with the station leader and the MD chief	Hospitalisation— Consider evacuation with the station leader and the MD chief
**Exams**	No need	No need	U + E, Blood gas	Blood gas, ECG, chest X-ray, POCUS lung (B lines)

## Data Availability

The original contributions presented in this study are included in the article. Further inquiries can be directed to the corresponding authors.

## Abbreviations

The following abbreviations are used in this manuscript:

AMSL	Above Mean Sea Level
AMS	Acute Mountain Sickness
bpm	Beats per minute
CPAP	Continuous Positive Airway Pressure
DC	Dome C
ECG	Electrocardiogram
EPAP	Expiratory Positive Airway Pressure
HACE	High-altitude cerebral oedema
HAPE	High altitude pulmonary oedema
PEEP	Positive End-Expiratory Pressure
NZ	New Zealand
PNRA	Programma Nazionale di Ricerche in Antartide
TAAF	Terres australes et antarctiques françaises
U + E	Urea + Electrolytes
